# Oral Supplementation of Phosphatidylcholine Attenuates the Onset of a Diet-Induced Metabolic Dysfunction–Associated Steatohepatitis in Female C57BL/6J Mice

**DOI:** 10.1016/j.jcmgh.2024.01.009

**Published:** 2024-01-21

**Authors:** Victor Sánchez, Anja Baumann, Annette Brandt, Maximilian F. Wodak, Raphaela Staltner, Ina Bergheim

**Affiliations:** Department of Nutritional Sciences, Molecular Nutritional Science, University of Vienna, Vienna, Austria

**Keywords:** Phosphatidylcholine, Inflammation, MASLD, Peroxisome Proliferator-Activated Receptor γ, NFκB

## Abstract

**Background & Aims:**

Changes in phosphatidylcholine levels in the liver have been associated with the development of metabolic dysfunction–associated steatotic liver disease. Here, the effects of supplementing phosphatidylcholine on the development of early signs of metabolic dysfunction–associated steatohepatitis were assessed.

**Methods:**

Male and female C57BL/6J mice were fed a liquid control or a fructose-, fat-, and/or cholesterol-rich diet for 7 or 8 weeks. The diets of female mice were fortified ± phosphatidylcholine (12.5 mg/g diet). In liver tissue and portal blood, indices of liver damage, inflammation, and bacterial endotoxemia were measured. J774A.1 cells and human monocytes preincubated with phosphatidylcholine (0.38 mmol/L) were challenged with lipopolysaccharide (50–100 ng/mL) ± the peroxisome proliferator-activated receptor γ (PPARγ) activator pioglitazone (10 μmol/L) or ± a liver receptor homolog 1 (LRH-1) antagonist 1-(3′-[1-(2-[4-morpholinyl]ethyl)-1H-pyrazol-3-yl]-3-biphenylyl)ethanon (1–10 μmol/L).

**Results:**

In fructose-, fat-, and/or cholesterol-rich diet–fed mice the development of fatty liver and the beginning of inflammation were associated with significantly lower hepatic phosphatidylcholine levels when compared with controls. Supplementing phosphatidylcholine significantly attenuated the development of fatty liver and inflammation, being associated with protection against the induction of PPARγ2, and activation of nuclear factor of κ light polypeptide gene enhancer in B-cell inhibitor α whereas *Lrh1* expression was unchanged. The protective effects of phosphatidylcholine on the lipopolysaccharide-induced activation of J774A.1 cells and human monocytes were attenuated significantly by the PPARγ activator pioglitazone and the LRH-1 antagonist.

**Conclusions:**

Our data suggest that phosphatidylcholine levels in the liver are lower in early metabolic dysfunction–associated steatohepatitis in mice and that supplementation of phosphatidylcholine can diminish the development of metabolic dysfunction–associated steatotic liver disease through mechanisms involving LRH-1/PPARγ2/ nuclear factor κ-light-chain enhancer of activated B-cell signaling.

## Graphical abstract

**Figure:**
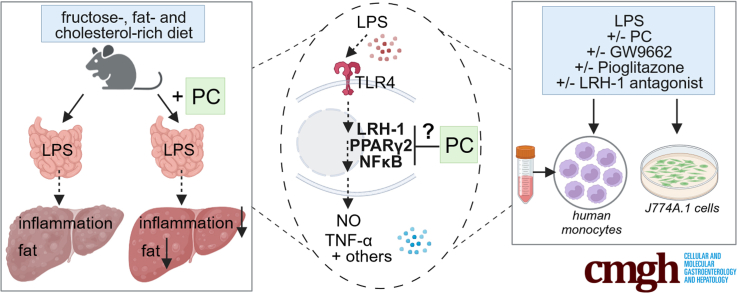

**Summary**

Diet-induced metabolic dysfunction–associated steatotic liver disease is associated with lower phosphatidylcholine levels in the liver. Supplementing phosphatidylcholine protected against fatty liver and inflammation through mechanisms involving alterations of the liver receptor homolog 1/peroxisome proliferator-activated receptor γ 2 and nuclear factor κ-light-chain enhancer of activated B-cell signaling in the liver.

With a still-increasing prevalence, metabolic dysfunction–associated steatotic liver disease (MASLD), until recently referred to as nonalcoholic fatty liver disease,^1^ by now is the most prevalent liver disease worldwide. Indeed, it is estimated that approximately 30% of the general population worldwide is afflicted with MASLD.^2^ Ranging from simple steatosis and steatohepatitis (metabolic dysfunction–associated steatohepatitis [MASH]) to fibrosis and even cirrhosis, MASLD comprises a wide range of disease.^3^ Besides age, genetic predisposition, and general overnutrition, in more recent years, several dietary factors such as a diet rich in saturated fatty acids and sugars have been associated with the development of MASLD.^4^^,^^5^ The resulting chronic excessive inflow of lipids and enhanced de novo lipogenesis is by now well acknowledged as one factor in the multifactorial development of MASLD (for overview, see Hughey et al^6^). Furthermore, changes of intestinal microbiota composition and an increased translocation of lipopolysaccharide (LPS) subsequently leading to activation of Toll-like receptor 4 (TLR4) depending signaling cascades have been shown repeatedly to be associated with the development of MASLD,^7^, ^8^, ^9^, ^10^, ^11^ and also may interact with hepatic lipid homeostasis.^12^ Although some progress in understanding has been made, to date the molecular mechanisms associated with the development of MASLD are not fully understood and universally accepted therapies are lacking.

Phosphatidylcholine comprises approximately 40%–50% of total cellular phospholipids and is the most abundant phospholipid in mammalian cells and subcellular organelles (for overview, see van der Veen et al^13^). Changes in the hepatic phosphatidylcholine level and especially in the phosphatidylcholine/phosphatidylethanolamine ratio have been linked repeatedly to the development of MASLD in both human beings^14^ and rodent models of the disease.^15^, ^16^, ^17^ Herein, impairments of very low density lipoprotein (VLDL) are discussed to play a pivotal role^15^; however, it also has been suggested that a lack of phosphatidylcholine may enhance inflammatory processes through VLDL-independent mechanisms.^16^ Specifically, it has been suggested that phosphatidylcholine can alter macrophage polarization and reduce the inflammatory response, for example, LPS-induced expression of proinflammatory cytokines such as tumor necrosis factor α (TNF-α)^18^ through as yet not fully determined mechanisms.

Starting from this background, the aim of the present study was to assess if an oral supplementation of phosphatidylcholine prevents the onset of an early diet-induced MASH in mice and to determine the molecular mechanisms involved.

## Results

### Phosphatidylcholine Levels in Livers of Fructose-, Fat-, and Cholesterol-Rich Diet–Fed Female and Fructose- and Fat-Rich Diet–Fed Male Mice

**Figure 1:**
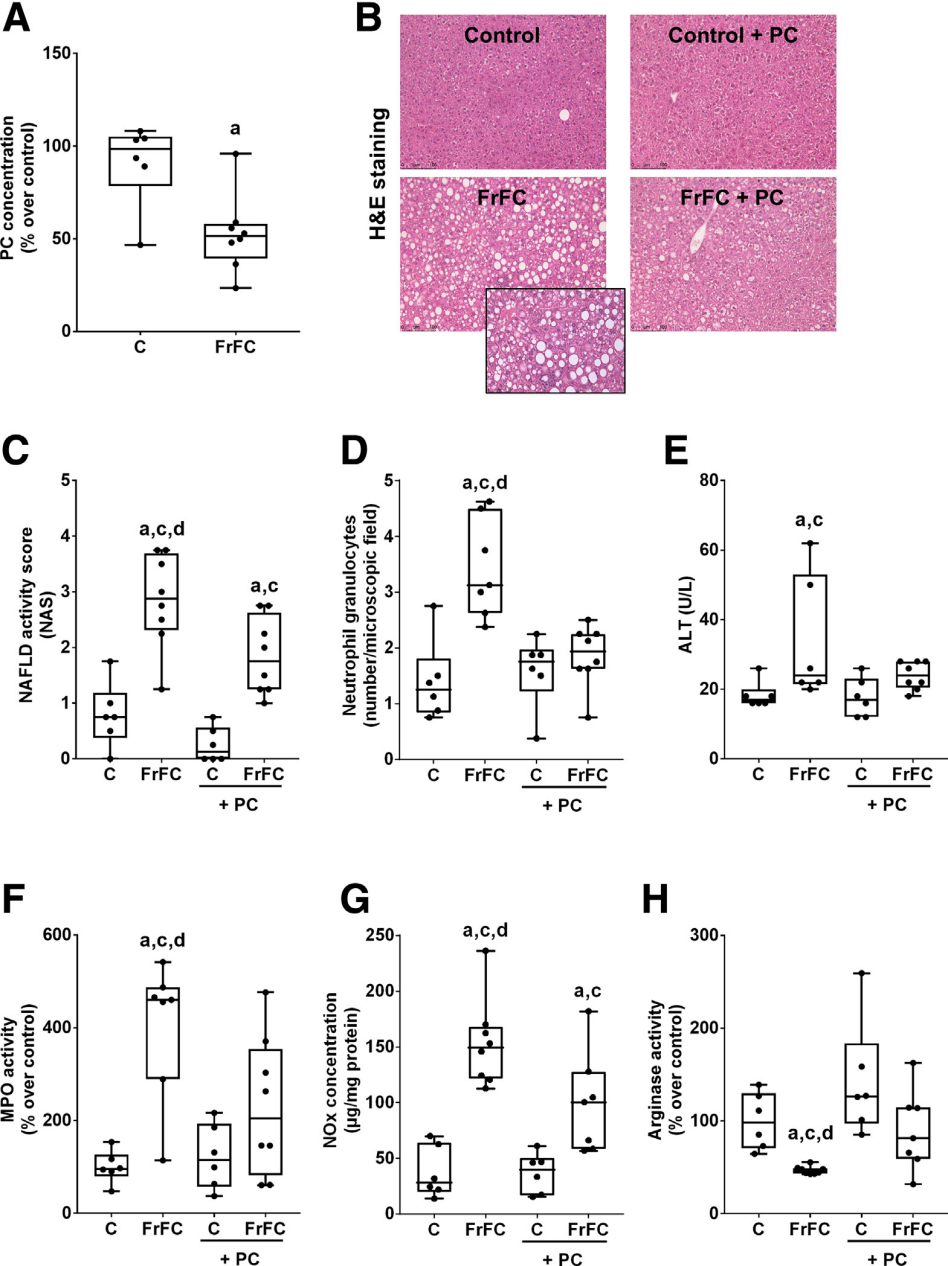
**Effect of supplementing phosphatidylcholine on markers of liver damage in female C57BL/6J mice fed a FrFC diet.** (*A*) PC in liver tissue. (*B*) Representative pictures of H&E staining in liver tissue (magnification, 200× and 400×). (*C*) NAS of liver sections. (*D*) Number of neutrophil granulocytes per microscopic field in liver tissue. (*E*) Alanine aminotransferase (ALT) activity in plasma. (*F*) MPO activity. (*G*) NO concentration. (*H*) Arginase activity in liver tissue. Data are presented as box and whisker plots, n = 6–8 mice per group. ^a^*P* < .05 compared with mice fed the control diet. ^c^*P* < .05 compared with mice fed the control+phosphatidylcholine diet. ^d^*P* < .05 compared with mice fed the FrFC+phsophatidylcholine diet. NAFLD, nonalcoholic fatty liver disease.

**Figure 2:**
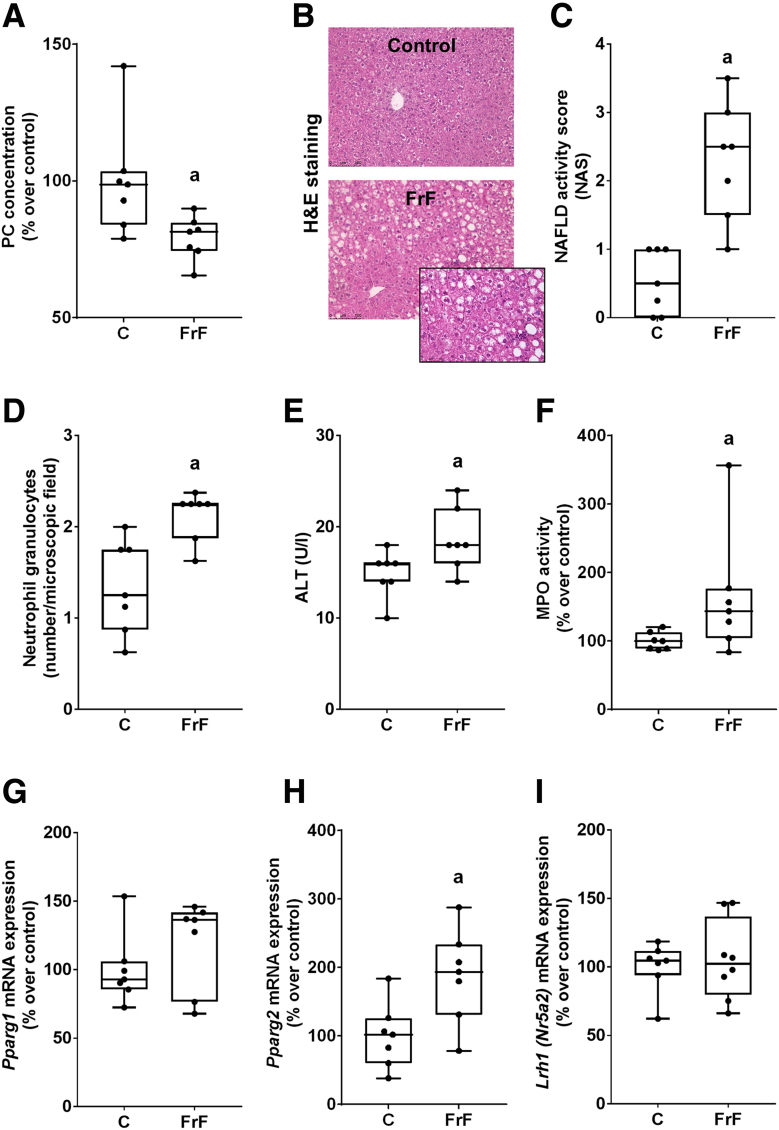
**Phosphatidylcholine levels, markers of liver damage, and PPARγ1 and 2 in livers of male C57BL/6J mice fed a FrF diet.** (*A*) Hepatic PC concentration. (*B*) Representative picture (magnification, 200× and 400×) of H&E staining. (*C*) NAS of liver sections. (*D*) Number of neutrophil granulocytes per microscopic field in livers. (*E*) Alanine aminotransferase (ALT) activity in plasma. (*F*) MPO activity in liver tissue. Hepatic mRNA expression of (*G*) *Pparg1* and (*H*) *Pparg2*, as well as (*I*) *Lrh1* (*Nr5a2*). Data are expressed as box and whisker plots, n = 7 mice per group. ^a^*P* < .05. Lrh1 (Nr5a2), liver receptor homolog 1.

As expected, mice fed a MASLD-inducing diet (eg, a fructose-, fat-, and cholesterol-rich [FrFC] or fructose- and fat-rich [FrF] diet), regardless of sex, developed early signs of MASH, as seen by a significantly higher nonalcoholic fatty liver disease activity score (NAS), alanine aminotransferase activity, and numbers of neutrophil granulocytes when compared with control diet–fed mice. Also, lymphocyte antigen 6 complex locus G6D (Ly6G)-positive cells and myeloperoxidase (MPO) activity were significantly higher in livers of male and female mice fed the MASLD-inducing diets when compared with control diet–fed mice (Figures 1*B–F* and 2*B–F*, and Tables 1 and 2). The development of early MASH in both male and female mice fed a MASLD-inducing diet was associated with a significantly lower phosphatidylcholine concentration in liver tissue compared with control diet–fed mice (*P* < .05) (Figures 1*A* and 2*A*).

**Table 1:** Effect of Supplementing Phosphatidylcholine on Food Intake, Body Weight, Glycemia, and Triglyceride Concentration in Liver Tissue in Female C57BL/6J Mice Fed a FrFC Diet

Parameters	Diet groups			
	C	FrFC	C+PC	FrFC+PC
Food intake, kcal/g body weight/d	0.40 ± 0.08	0.45 ± 0.05^a^^,^^c^	0.39 ± 0.07	0.44 ± 0.06^a^^,^^c^
Body weight, *g*	22.1 ± 1.5	22.5 ± 1.1	22.5 ± 1.6	22.6 ± 1.5
Liver weight, *g*	1.0 ± 0.2	1.5 ± 1.0^a^^,^^c^	1.1 ± 0.1	1.4 ± 0.1^a^^,^^c^
Liver:body weight ratio, *%*	4.4 ± 0.7	6.7 ± 0.3^a^^,^^c^^,^^d^	4.9 ± 0.5	6.0 ± 0.3^a^^,^^c^
Steatosis, NAS	0.2 ± 0.2	2.2 ± 0.7^a^^,^^c^	0.2 ± 0.2	1.7 ± 0.7^a^^,^^c^
Inflammation, NAS	0.5 ± 0.4	0.7 ± 0.3^c^^,^^d^	0.1 ± 0.1	0.2 ± 0.2
Ly6G staining, number of positive cells	1.2 ± 0.6	4.8 ± 3.4^a^^,^^d^	1.8 ± 1.1	1.6 ± 0.1
Sirius red staining, % of microscopic field	0.05 ± 0.02	0.09 ± 0.17	0.03 ± 0.02	0.02 ± 0.02
Glucose, AUC	25,282 ± 3220	29,421 ± 2672	28,701 ± 3883	31,914 ± 6183
Fasting blood glucose level, *mg/dL*	103 ± 14	108 ± 13	108 ± 10	111 ± 10
Triglyceride, % over control	100 ± 41	366 ± 203^a^^,^^c^	124 ± 73	284 ± 68^a^^,^^c^

NOTE. Data are presented as means ± SD, n = 6–8 mice per group.

AUC, area under the curve.

a *P* < .05 compared with mice fed the control diet.

c *P* < .05 compared with mice fed the control+phosphatidylcholine diet.

d *P* < .05 compared with mice fed the FrFC+phosphatidylcholine diet.

**Table 2:** Food Intake, Body Weight, and Liver Damage in Male C57BL/6J Mice Fed a FrF Diet

Parameters	Diet groups	
	C	FrF
Food intake, kcal/g body weight/d	0.37 ± 0.1	0.44 ± 0.1^a^
Body weight, *g*	27.2 ± 1.1	27.9 ± 1.9
Liver weight, *g*	1.13 ± 0.04	1.44 ± 0.3^a^
Liver:body weight ratio, *%*	4.14 ± 0.2	5.14 ± 0.7^a^
Steatosis, NAS	0.30 ± 0.39	1.29 ± 0.57^a^
Inflammation, NAS	0.25 ± 0.38	1.00 ± 0.50^a^
Ly6G staining, number of positive cells	1.2 ± 0.7	2.1 ± 0.9^a^

NOTE. Data are presented as means ± SD, n = 7 mice per group.

a *P* < .05.

### Effects of Supplementing Phosphatidylcholine on Markers of Liver Damage in FrFC-Fed Female Mice

Despite similar caloric intake and body weight (Table 1), NAS and herein especially signs of hepatic inflammation such as the number of neutrophil granulocytes, MPO activity, and Ly6G-positive cells were significantly lower in livers of FrFC-fed mice treated concomitantly with phosphatidylcholine when compared with FrFC-fed mice (*P* < .05 for all parameters) (Table 1 and Figure 1*C*, *D*, and *F*). In contrast, steatosis scoring and hepatic triglyceride levels, as well as alanine aminotransferase activity in plasma, were similar between FrFC-fed groups regardless of additional treatment (Table 1 and Figure 1*E*). However, NAS and liver to body weight ratios of FrFC+phosphatidylcholine–fed mice still were significantly higher than in control diet–fed groups (*P* < .05) (Figure 1*C* and Table 1). Liver to body weight ratios also were significantly lower in FrFC+phosphatidylcholine–fed mice when compared with FrFC-fed mice (*P* < .05) (Table 1). Applying Sirius red staining, no signs of fibrosis were detected in any of the groups (Table 1). Nitric oxide (NO) concentration in liver tissue was significantly higher in livers of FrFC-fed mice compared with all other groups (*P* < .05) (Figure 1*G*). NO concentrations in livers of FrFC+phosphatidylcholine–fed mice still were significantly higher than in control diet–fed groups (*P* < .05) (Figure 1*G*). Arginase activity, suggested to be the counterplayer of inducible nitric oxide synthase,^19^ also was significantly lower in livers of FrFC-fed mice compared with all other groups (*P* < .05) (Figure 1*H*). No differences in arginase activity were found between FrFC+phosphatidylcholine–fed mice and the 2 control groups (Figure 1*H*). Because mice did not show signs of overweight and only early signs of MASH, no differences in markers of glucose metabolism were found between groups (Table 1).

### Effect of Supplementing Phosphatidylcholine on Apolipoprotein B Expression in Liver Tissue of FrFC-Fed Mice

**Figure 3:**
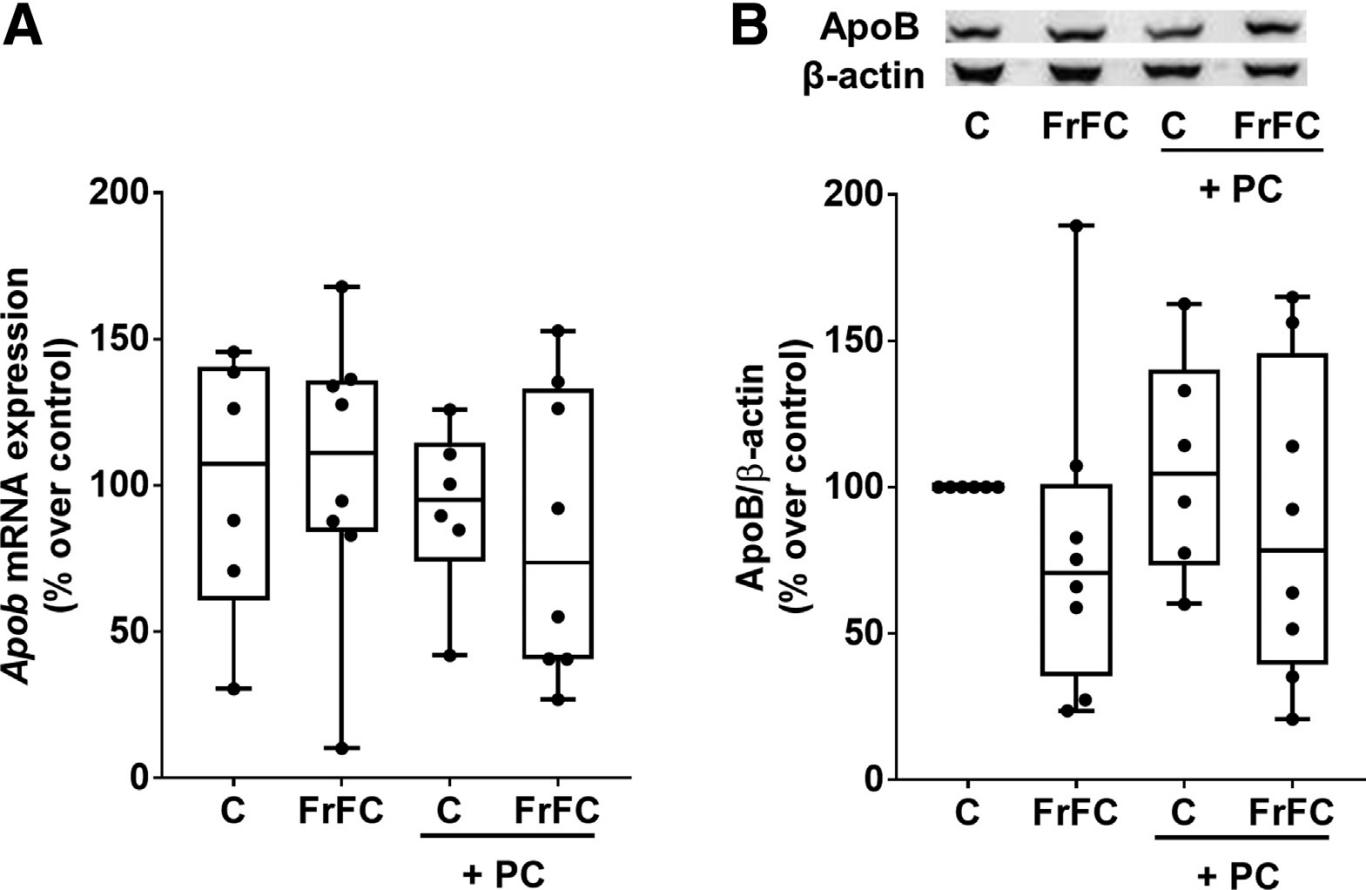
**Effect of supplementing phosphatidylcholine on apolipoprotein B mRNA and protein levels in liver tissue of female C57BL/6J mice fed a FrFC diet.** (*A*) Expression of apolipoprotein B (*Apob*) mRNA. (*B*) Hepatic protein concentration of ApoB normalized to β-actin. Data are presented as box and whisker plots, n = 6–8 mice per group.

Because it has been suggested that phosphatidylcholine may alter VLDL secretion through apolipoprotein B (ApoB)-dependent mechanisms,^15^^,^^20^ we next determined if the protective effects found for the supplementation of phosphatidylcholine were associated with changes in ApoB expression in liver. Neither mRNA expression of *Apob* nor protein levels in liver tissue differed between groups (Figure 3).

### Effect of Supplementing Phosphatidylcholine on TLR4-Dependent Signaling Cascades and Peroxisome Proliferator-Activated Receptor γ 1 and 2 and Liver Receptor Homolog 1 in Liver Tissue of Female FrFC-Fed Mice and *Pparg1* and *2* as Well as *Lrh1* mRNA Expression in Livers of Male FrF-Fed Mice

**Figure 4:**
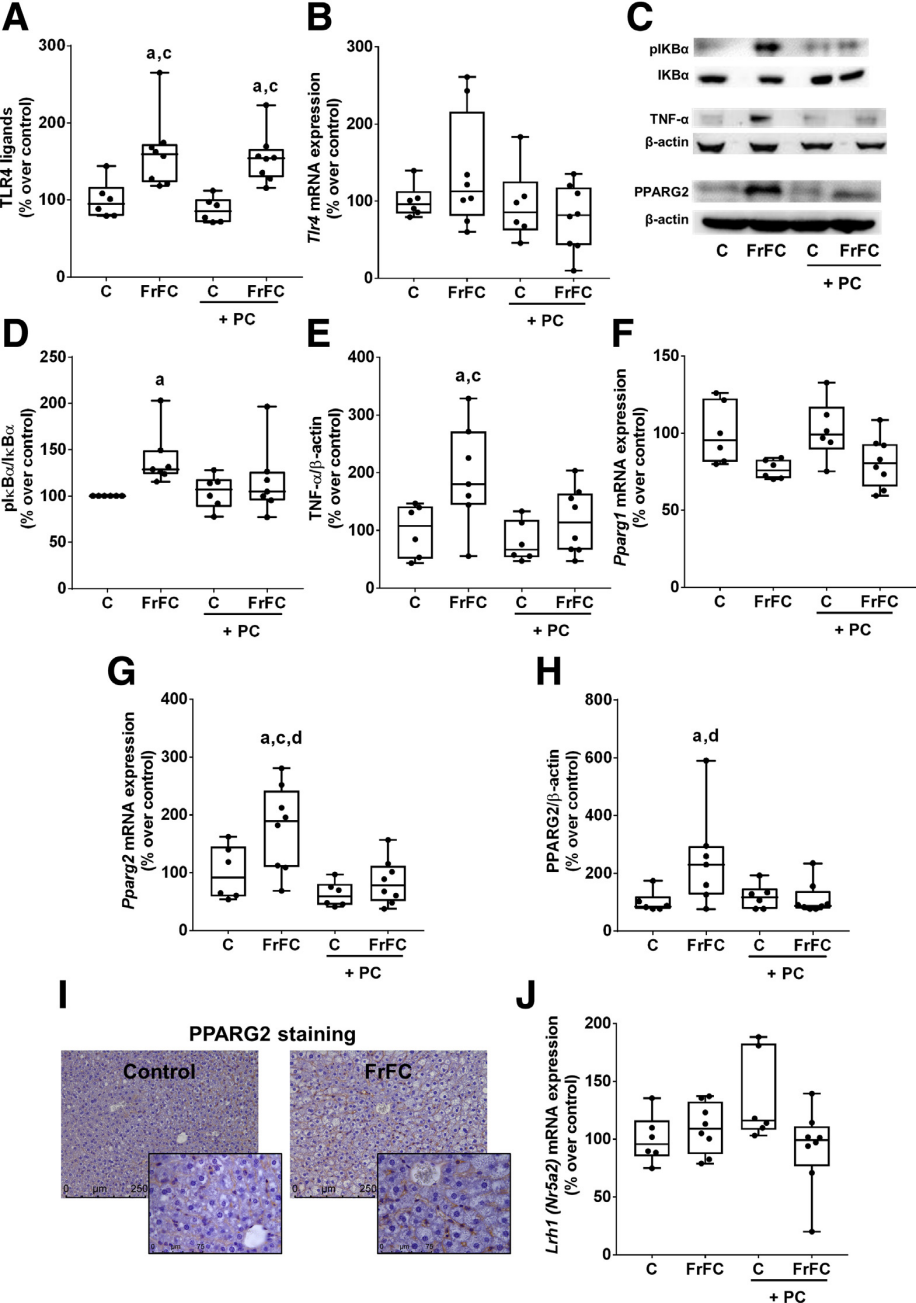
**Effect of supplementing phosphatidylcholine on TLR4 ligands in portal blood, and expression of *Tlr4*, TNF-α*, Pparγ1* and *2*, as well as activation of IkBα in liver tissue in female C57BL/6J mice fed a FrFC diet.** (*A*) Levels of TLR4 ligands in plasma. (*B*) mRNA expression of *Tlr4* in liver tissue. (*C*) Representative pictures of Western blots. (*D*) Hepatic protein concentration of phospho-IkBα. (*E*) Hepatic protein concentration of TNF-α. Hepatic mRNA expression of (*F*) *Pparg1* and (*G*) *Pparg2*. (*H*) Hepatic protein concentration of PPARG2 normalized to β-actin. (*I*) Representative pictures of PPARG2 staining (magnification, 400× and 630×). (*J*) Hepatic mRNA expression of *Lrh1* (*Nr5a2*). Data are presented as box and whisker plots, n = 6–8 mice per group. ^a^*P* < .05 compared with mice fed the control diet. ^c^*P* < .05 compared with mice fed the control+phosphatidylcholine diet. ^d^*P* < .05 compared with mice fed the FrFC+phosphatidylcholine diet. Lrh1 (Nr5a2), liver receptor homolog 1.

It has been suggested that phosphatidylcholine may improve intestinal barrier function^21^^,^^22^ and that an increased intestinal translocation of LPS leading to an activation of TLR4 and subsequently an activation of nuclear factor κ-light-chain enhancer of activated B cells (NFκB)-dependent signaling may be critical in the development of MASLD.^7^^,^^8^^,^^23^ Therefore, concentration of TLR4 ligands (LPS) in portal plasma and mRNA expression of *Tlr4* and dependent signaling cascades in liver tissue were determined. Concentrations of TLR4 ligands (LPS) were significantly higher in portal plasma of both FrFC-fed groups than in control groups (*P* < .05) (Figure 4*A*), and were similar between FrFC-fed groups (Figure 4*A*). Expression of *Tlr4* mRNA in liver tissue did not differ between groups because expression varied considerably within groups (Figure 4*B*). Phosphorylation of nuclear factor of κ light polypeptide gene enhancer in B-cell inhibitor (IκBα) in liver tissue being indicative of NFκB activation^24^ also was significantly higher in livers of FrFC-fed mice when compared with control diet–fed group (*P* < .05) (Figure 4*D*), while being almost at the level of controls in FrFC+phosphatidylcholine–fed groups (Figure 4*D*). In line with these findings, TNF-α protein concentration was significantly (control diet [C] vs FrFC and C+phosphatidylcholine vs FrFC), and by trend, higher in livers of FrFC-fed mice than in FrFC+phosphatidylcholine–fed mice (FrFC vs FrFC+phosphatidylcholine: *P* = .09) (Figure 4*E*). Because results of studies suggest that phosphatidylcholine may regulate peroxisome proliferator-activated receptor γ (PPARγ) and that PPARγ may alter NFκB activation,^25^^,^^26^ we next determined the mRNA expression of the 2 splice variants of PPARγ: *Pparg1* and *2*. Expression of *Pparg1* mRNA expression was similar between groups (Figure 4*F*). In contrast, *Pparg2* mRNA expression was significantly higher in livers of FrFC-fed mice than in all other groups (*P* < .05) (Figure 4*G*). In line with these findings, PPARG2 protein levels in liver homogenate were significantly higher in livers of FrFC-fed mice compared with control diet–fed and FrFC+phosphatidylcholine–fed animals (Figure 4*H*). In livers of FrFC+phosphatidylcholine–fed animals PPARG2 protein concentration was almost at the level of controls (Figure 4*H*). Immunohistochemical staining of liver sections with a specific PPARγ2 antibody revealed that this splice variant of the nuclear receptor is not detectable in hepatocytes, but rather in immune cells (eg, Kupffer cells) found in liver tissue (representative pictures of staining are shown in Figure 4*I*). In contrast, expression of *Lrh1* (*Nr5a2*) mRNA, suggested to be regulated by certain phosphatidylcholines,^27^ did not differ between groups (Figure 4*J*).

### Effects of Supplementing Phosphatidylcholine on LPS-Dependent Activation of J774A.1 Cells and Primary Human Monocytes Isolated From Peripheral Blood

**Figure 5:**
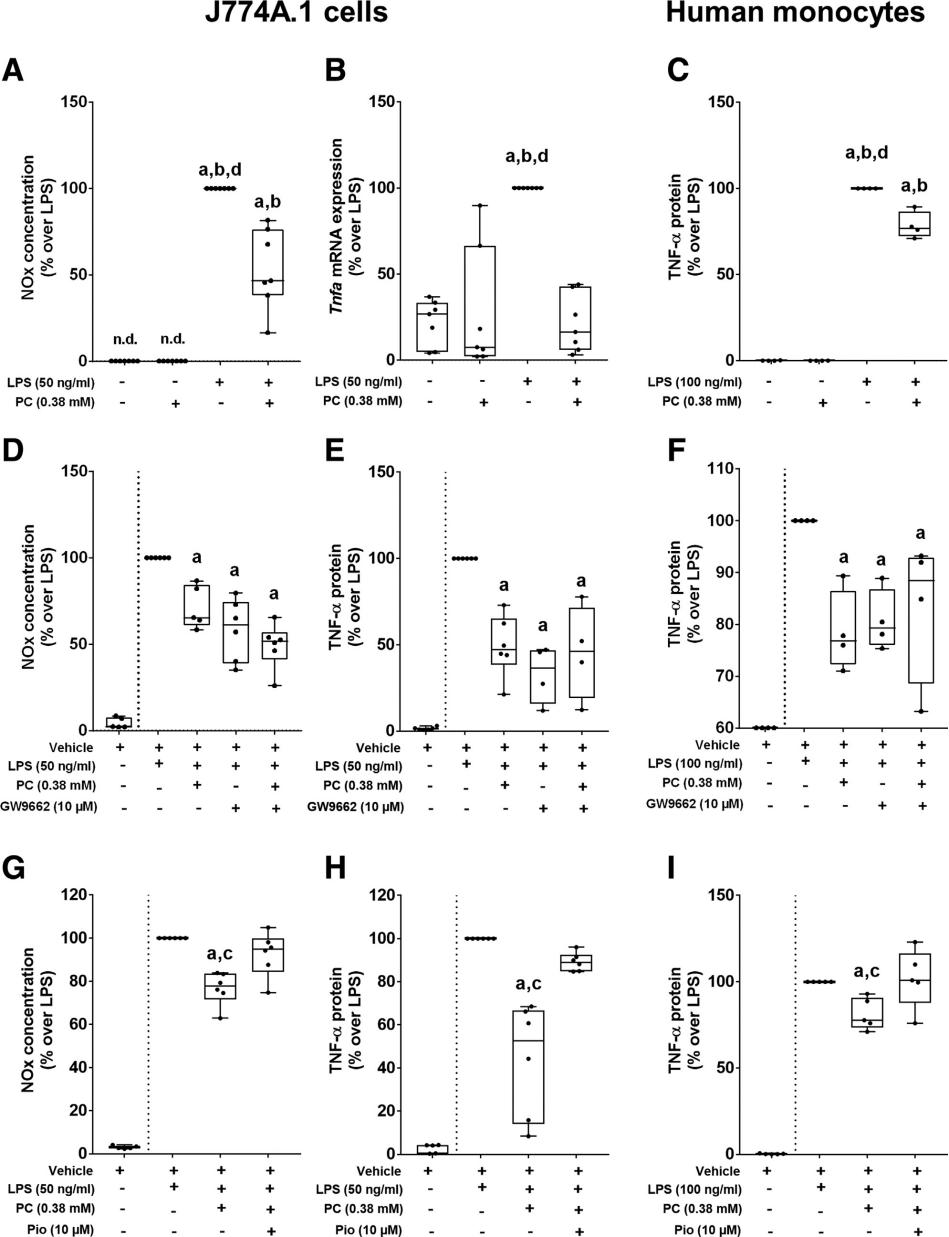
**Effect of phosphatidylcholine on the LPS-induced activation of J774A.1 cells and primary human monocytes isolated from buffy coats of healthy donors.** (*A*) NO concentration in cell culture medium. (*B*) mRNA expression of *Tnfa* in stimulated J774A.1 cells treated with phosphatidylcholine. (*C*) TNF-α protein in supernatant of LPS-stimulated human monocytes treated with phosphatidylcholine. (*D*) NO concentration and (*E*) TNF-α protein concentration in supernatant of LPS-stimulated J774A.1 cells treated with phosphatidylcholine and GW9662. (*F*) TNF-α protein concentration in cell culture media of LPS-stimulated human monocytes treated with phosphatidylcholine and GW9662. (*G*) NO concentration and (*H*) TNF-α protein concentration in cell culture media of LPS-stimulated J774A.1 cells treated with phosphatidylcholine and pioglitazone. (*I*) TNF-α protein concentration in LPS-stimulated human monocytes treated with phosphatidylcholine and pioglitazone. Data are presented as box and whisker plots, n = 4–7. (*A* and *B*) n = 7. ^a^*P* < .05 compared with naïve J774A.1 cells. ^b^*P* < .05 compared with J774A.1 cells treated with phosphatidylcholine. ^d^*P* < .05 compared with J774A.1 cells treated with LPS+phosphatidylcholine. (*C*) n = 4. ^a^*P* < .05. compared with naïve human monocytes. ^b^*P* < .05 compared with human monocytes treated with phosphatidylcholine. ^d^*P* < .05 compared with human monocytes treated with LPS+phosphatidylcholine. (*D* and *E*) n = 6. ^a^*P* < .05 compared with J774A.1 cells treated with LPS. (*F*) n = 4. ^a^*P* < .05 compared with human monocytes treated with LPS. (*G* and *H*) n = 6. ^a^*P* < .05 compared with J774A.1 cells treated with LPS. ^c^*P* < .05 compared with J774A.1 cells treated with LPS+phosphatidylcholine+pioglitazone. (*I*) n = 5. ^a^*P* < .05 compared with human monocytes treated with LPS. ^c^*P* < .05 compared with human monocytes treated with LPS+phosphatidylcholine+pioglitazone. n.d., not detectable; NOx, nitric oxide; Pio, pioglitazone.

To further delineate the mechanisms related to the effects of phosphatidylcholine on the LPS-induced inflammatory response, J774A.1 cells used as a model of Kupffer cells as well as primary monocytes isolated from buffy coats of healthy donors were stimulated with LPS in the presence or absence of phosphatidylcholine. As expected, concentrations of NO in medium and mRNA expression of *Tnfa* both were significantly higher in J774A.1 cells treated with LPS when compared with control groups (*P* < .05) (Figure 5*A* and *B*). The preincubation with phosphatidylcholine significantly blunted the LPS-induced *Tnfa* mRNA expression (Figure 5*B*) and NO formation (*P* < .05) (Figure 5*A*); however, NO levels still were higher in cell culture medium of LPS+phosphatidylcholine–treated cells than in both control groups (*P* < .05) (Figure 5*A*). In human monocytes, TNF-α protein concentration was significantly higher in LPS-challenged cells compared with control groups and LPS+phosphatidylcholine–treated cells (Figure 5*C*). Although significantly lower than in LPS-challenged monocytes, TNF-α protein levels still were higher in LPS-phosphatidylcholine–treated cells than in both control groups (Figure 5*C*).

### Effects of Phosphatidylcholine on LPS-Induced Activation of J774A.1 Cells and Human Monocytes Preincubated With a PPARγ Agonist and an Antagonist, Respectively

To further delineate the molecular mechanisms underlying the protective effects of phosphatidylcholine, and to delineate if PPARγ2 might be critical herein, J774A.1 cells and human primary monocytes, respectively, were either preincubated with phosphatidylcholine, GW9662, an antagonist of PPARγ shown to attenuate the induction of PPARγ2 in mice with diet-induced MASLD,^10^ or the PPARγ activator pioglitazone. Both pretreating J774A.1 cells and human monocytes with phosphatidylcholine and GW9662 significantly attenuated the LPS-dependent increase of NO and TNF-α protein levels in cell culture medium (*P* < .05) (Figure 5*D–F*). When cells were pretreated with a combination of phosphatidylcholine and GW9662, the effects were similar to the effects found for either compound alone (Figure 5*D–F*). In contrast, preincubating J774A.1 cells and human monocytes, respectively, with phosphatidylcholine and the PPARγ agonist, pioglitazone, abolished the protective effects of phosphatidylcholine on the release of NO and TNF-α protein levels into the cell culture media (Figure 5*G–I*).

### Effects of Phosphatidylcholine on LPS-Induced Activation of J774A.1 Cells or Primary Human Monocytes Preincubated With an Antagonist of Liver Receptor Homolog 1 (NR5A2)

**Figure 6:**
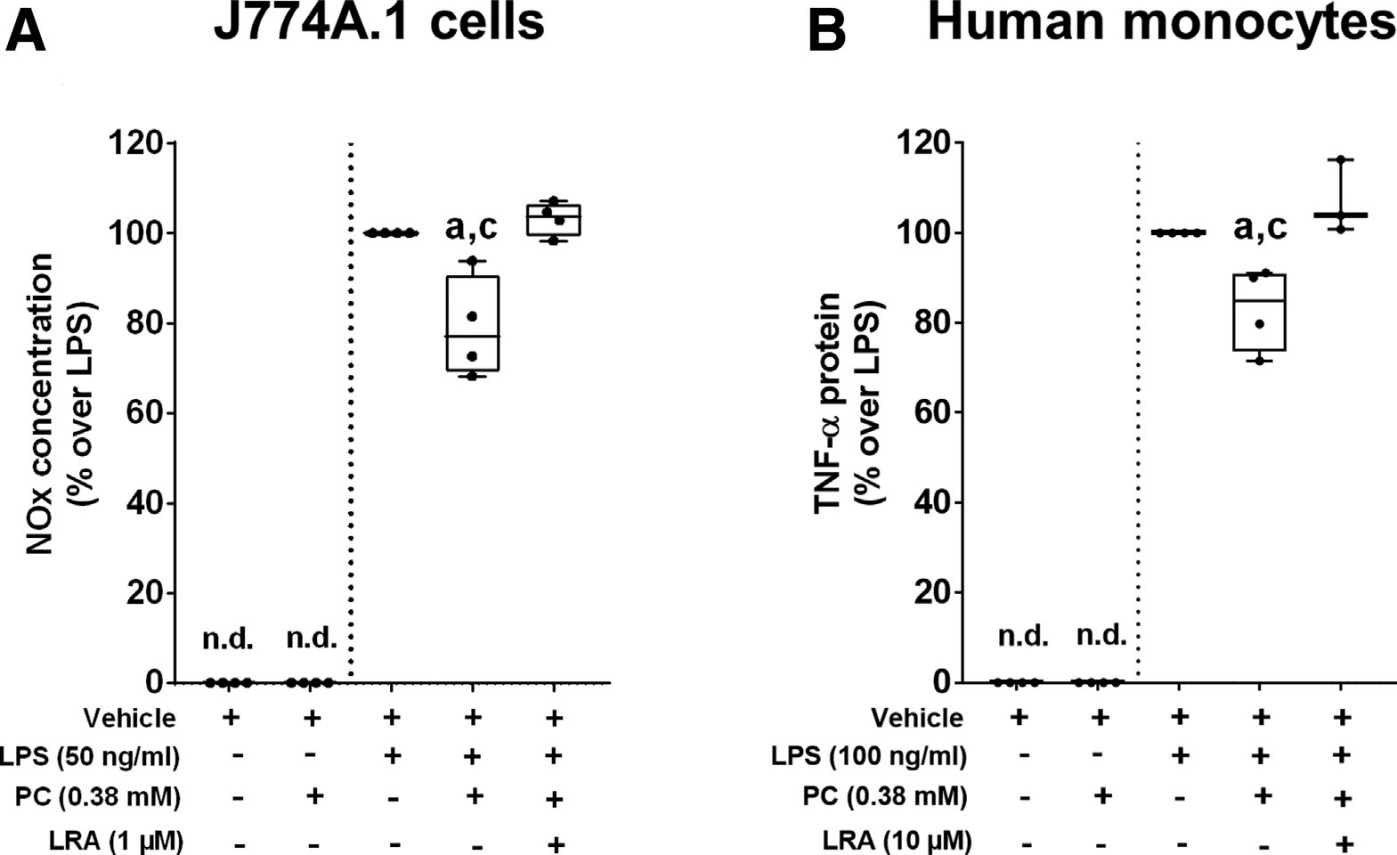
**Effect of phosphatidylcholine and an antagonist of LRH-1 on LPS-induced activation of J774A.1 cells and human primary monocytes isolated from buffy coats of healthy donors.** (*A*) NO concentration in cell culture medium. (*B*) TNF-α protein concentration in cell culture media of LPS-stimulated cells treated with phosphatidylcholine and the antagonist of LRH-1. Data are presented as box and whisker plots. n = 4. ^a^*P* < .05 compared with cells treated with LPS. ^c^*P* < .05 compared with cells treated with LPS+phosphatidylcholine+LRH-1 antagonist (1-[3’-(1-[2-(4-morpholinyl)ethyl]-1H-pyrazol-3-yl)-3-biphenylyl]ethanon). LRA, antagonist of LRH-1; n.d., not detectable; NOx, nitric oxide.

Because activation of liver receptor homolog 1 (LRH-1) has been suggested as a potential target for certain phosphatidylcholines,^28^ J774A.1 cells and primary human monocytes, respectively, were either preincubated with phosphatidylcholine or an antagonist of LRH-1. The antagonist of LRH-1 significantly abolished the protective effects of phosphatidylcholine on the release of NO and TNF-α protein levels into the cell culture media (Figure 6).

## Discussion

Results of both human and animal studies have suggested that the development of MASLD is associated with lower levels of phosphatidylcholine.^14^, ^15^, ^16^, ^17^^,^^29^ In line with these findings and despite being fed sufficient amounts of choline, female and male mice fed MASLD-inducing diets had significantly lower hepatic phosphatidylcholine levels when compared with mice fed a control diet in the present study. These lower levels of phosphatidylcholine in liver tissue were associated with the development of hepatic macrovesicular steatosis and signs of liver inflammation. In livers of female mice receiving the FrFC diet enriched with phosphatidylcholine, these alterations were attenuated markedly, with most inflammatory markers being almost at the level of controls. Because mice still were of normal weight and were only fed the FrFC diet for 8 weeks, no alterations in glucose tolerance were observed yet. Indeed, we showed before that when using this model of lean MASLD, the development of alterations in glucose metabolism and insulin resistance might take longer than in models of overweight-associated MASLD.^10^^,^^30^^,^^31^ Further suggesting that in the present study phosphatidylcholine predominantly affected inflammatory alterations, NO levels also were almost at the level of controls in livers of female mice fed the FrFC diet supplemented with phosphatidylcholine. NO has been reported before to be indicative of an induction of inducible nitric oxide synthase in settings of MASLD,^32^ and also M1 polarization.^33^ In line with these findings, arginase activity, also discussed to be an indicator of macrophage polarization,^34^ was lower in livers of female FrFC-fed mice. Again, these alterations were abolished almost completely in livers of female mice fed the FrFC diet fortified with phosphatidylcholine. These findings are also in line with those of others proposing that a lack of phosphatidylcholine may add to the development of proinflammatory alterations in settings of MASLD.^13^^,^^35^ Interestingly, and somewhat contrasting the findings of the present study suggesting that phosphatidylcholine derived from egg lecithin may especially attenuate the progression of steatosis to MASH, results of Niebergall et al,^15^ using liver-specific elimination of phosphocholine cytidyltransferase knock-out mice (LCTα^-/-^), suggested that normalizing hepatic phosphatidylcholine levels may not attenuate disease progression to inflammatory stages but may only affect the development of liver steatosis. Differences between the present study and that of Niebergall et al^15^ may have resulted from differences in feeding protocols, for example, 7 days of a high-fat diet vs 8 weeks of a high-fructose, high-fat, and cholesterol-rich diet, but also suggest that other mechanisms besides being a precursor for the synthesis of betaine may be critical in the beneficial effects of phosphatidylcholine in the setting of MASLD (see later).^15^

In summary, our results suggest that the development of MASLD is related to a decrease in phosphatidylcholine levels in liver tissues of male and female mice, even in a nonmalnutrition state. Our results also suggest that enriching the diet with phosphatidylcholine may diminish the development of MASLD in female mice and that this is related predominantly to a suppression of inflammatory alterations. However, if similar protective effects also are found in male mice and later stages of the disease, as well as in human beings, remain to be determined. Also, it remains to be determined if phosphatidylcholine from other sources than egg lecithin, having shown to have higher phosphatidylcholine levels (∼3 times higher) when compared with soy lecithin^36^, has similar beneficial effects on the development of diet-induced MASLD.

### The Protective Effects of Phosphatidylcholine Are Not Related to Changes in ApoB Expression in Liver Tissue

Studies have suggested that phosphatidylcholine may be critical in the regulation of ApoB and VLDL-dependent hepatic lipid export.^37^ In the present study, protein levels of ApoB did not differ between the 2 FrFC groups. It has been suggested before by the results of others that the effects of phosphatidylcholine on ApoB might be sex- and diet-specific.^38^ Indeed, Noga et al^39^^,^^40^ showed that phosphatidylethanolamine N-methyltransferase catalyzes the conversion of phosphatidylethanolamine to phosphatidylcholine and that in female phosphatidylethanolamine N-methyltransferase knock-out mice fed a high-fat/high-cholesterol diet, VLDL secretion and plasma ApoB100 lipoprotein levels were maintained normally whereas both were diminished in male mice. In the present study, we only used female mice to assess the effects of fortifying the diet with phosphatidylcholine. It may be that when performing experiments alike in male mice, supplementing the diet with phosphatidylcholine might have altered ApoB levels in the liver. Also, we only determined ApoB levels by Western blot and did not perform functional assays to assess VLDL secretion.

### The Protective Effects of Phosphatidylcholine Are Not Related to Protection Against the Translocation of LPS but Protection Against the Activation of NFκB Signaling

It has been shown previously that the development of MASLD is associated with impairments of intestinal barrier function and an increased translocation of LPS, resulting in activation of TLR4-dependent signaling cascades in the liver.^41^ It also has been shown previously in settings of inflammatory bowel disease that phosphatidylcholine may attenuate inflammatory alterations in the gut and subsequently improve intestinal barrier function.^42^^,^^43^ In the present study, concentrations of TLR4 ligands, for example, LPS being indicative of intestinal barrier function, were significantly higher in portal blood of both FrFC-fed groups regardless of additional treatments. Differences between the results of our study and those of others might have been related to differences in concentrations of phosphatidylcholine used. Indeed, in the present study, mice received 12.5 mg phosphatidylcholine per gram diet while in the trial assessing the effects of phosphatidylcholine in models of inflammatory bowel disease up to 100 mg/kg body weight were used.^43^ Somewhat in contrast to our findings for TLR4 ligand concentration, *Tlr4* mRNA expression in liver tissue did not differ between groups. Indeed, it has been shown before by us and others that *Tlr4* expression in liver tissue is not always induced in the presence of its ligands.^9^ Still, supporting the findings for TLR4 ligand concentration, phosphorylation of IκBα being the inhibitor of NFκB,^44^ and TNF-α also were both at the level of controls in FrFC-fed mice concomitantly treated with phosphatidylcholine while being significantly induced in livers of FrFC-fed mice. Also, pretreating J774A.1 cells or human monocytes with phosphatidylcholine attenuated the induction of *Tnfa* mRNA and formation of NO or TNF-α protein, further supporting the hypothesis that phosphatidylcholine may interfere with TLR4–NFκB–dependent signaling in immune cells. Indeed, it has been suggested before by the results of others that phosphatidylcholine may interfere with the LPS–TLR–NFκB signaling cascade.^45^^,^^46^ However, results of these studies also have suggested that phosphatidylcholine may not directly interfere with NFκB but rather that other mechanisms may be involved. For instance, results of studies in mice have suggested that injecting adipose tissue of mice with phosphatidylcholine may down-regulate PPARγ expression in fatty tissue.^47^ Also, recent studies have reported that both docosahexaenoic acid–phosphatidylcholine and eicosapentanoic acid–phosphatidylcholine suppress NFκB-dependent signaling through enhancing levels of PPARγ,^48^ further suggesting that PPARγ may act as a counterplayer of NFκB signaling, thereby dampening proinflammatory responses.^49^ In contrast, in a recently published study, it was shown that a high-fat diet–induced MASLD was associated with an induction of PPARγ in liver tissue. This induction was dampened when phospholipase D1, an enzyme catalyzing the formation of phosphatidic acid, from phosphatidylcholine was knocked out.^50^ Furthermore, structural studies have shown that phospholipids may be potential ligands of LRH-1.^51^ Indeed, it was reported that treatment with the phospholipid dilauroyl phosphatidylcholine protected mice from the development of MASLD and insulin resistance through LRH-1–dependent mechanisms because the protective effects of dilauroyl phosphatidylcholine were lost in LRH-1^-/-^ mice.^27^ In the present study, *Pparg1* mRNA expression was found to be similar between groups, whereas *Pparg2* mRNA expression and PPARG2 protein concentrations were found to be induced in liver of FrFC-fed mice, and, herein, especially in immune cells, while not being detectable in hepatocytes. Furthermore, in livers of mice fed the FrFC diet supplemented with phosphatidylcholine, *Pparg2* mRNA and protein levels were almost at the level of controls. Somewhat contrasting the findings of others reporting a down-regulation of LRH-1 in patients with MASLD or MASH,^52^ in the present study, expression of *Lrh1* mRNA was similar in female and male mice, respectively, fed the MASLD-inducing diets and controls. Also, the addition of phosphatidylcholine had no effects of *Lrh1* expression in livers of female FrFC-fed mice. Supporting the hypothesis that phosphatidylcholine may have protected FrFC-fed mice through diminishing the activation of PPARγ2, pretreating LPS-stimulated cells with the PPARγ inhibitor GW9662 and phosphatidylcholine diminished the induction of NO production and TNF-α expression in J774A.1 cells or human monocytes to an almost similar extent. Also, the concomitant treatment of LPS-challenged cells with phosphatidylcholine and the PPARγ agonist pioglitazone abolished the protective effects of phosphatidylcholine on NO formation and TNF-α protein release into cell culture medium. These data contrast the findings of others discussed earlier.^48^^,^^49^ However, it has been shown before that an induction of PPARγ2 is associated with the development of MASLD in various rodent models^10^^,^^53^ and that targeting PPARγ2 with the PPARγ antagonist GW9662 may attenuate the development of MASLD.^10^^,^^54^ It also has been shown in these studies that the protective effects of the PPARγ inhibitor were associated with a marked protection against inflammatory alterations associated with the development of MASLD, for example, the induction of proinflammatory cytokines and an increase in NO in liver tissue and cell culture supernatants.^10^ Furthermore, results of the in vitro experiments of the present study also suggest that the beneficial effects of phosphatidylcholine may be related to LRH-1 because the protective effects of phosphatidylcholine on LPS-induced production of NO and proinflammatory cytokines were abolished almost completely when cells were treated concomitantly with an LRH-1 antagonist.

Taken together, our data suggest that although intestinal barrier function was disturbed in FrFC-fed mice, enriching the diet with phosphatidylcholine had no effect on these alterations but rather protected the liver from the development of MASLD through suppressing the LPS-dependent activation of PPARγ2 and, subsequently, NFκB in liver tissue. These results by no means preclude that at other concentrations or diseases of other etiologies (eg, inflammatory bowel disease) phosphatidylcholine may alter intestinal barrier dysfunction. Also, our data do not preclude that the formation of phosphatidic acid and a subsequent induction of PPARγ may be critical in lipid accumulation in settings of MASLD. Rather, our data suggest that in a setting of nonobese diet-induced MASLD, the beneficial effects of phosphatidylcholine may be related predominantly to a counterregulation of LPS–TLR4–PPARγ2–NFκB–dependent proinflammatory responses in the liver. The exact role of LRH-1 and its interplay with PPARγ2 and NFκB herein needs to be determined in future studies.

## Conclusions

Taken together, results of our study further bolster the hypothesis that alterations in hepatic phosphatidylcholine levels are critical in the development of MASLD and that supplementing phosphatidylcholine may attenuate or at least diminish the development of MASLD. Results of the present study further suggest that the protective effects of phosphatidylcholine are related to a protection of alterations in the LRH-1/PPARγ/NFκB signaling cascade in the liver. However, further studies are needed to determine exact molecular mechanisms underlying the interaction of phosphatidylcholine with these nuclear receptors as well as cell types involved. Further studies also are needed to determine if supplementation of phosphatidylcholine also attenuates the progression of MASLD in human beings, and if this also is related to changes in LRH-1/PPARγ2/NFκB signaling.

## Methods

### Animals and Treatment

Female and male C57BL/6J mice were obtained from Janvier (Janvier SAS, Le-Genest-Saint-Isle, France) and housed in a specific-pathogen-free barrier facility. All procedures were approved by the local institutional animal care and use committee (Bundesministerium für Wissenschaft, Forschung und Wirtschaft, Referat für Tierversuchswesen und Gentechnik, Vienna, Austria) and animals were handled in accordance with the European Convention for the Protection of Vertebrate Animals used for Experimental and Other Scientific Purposes. All experiments were performed under controlled conditions (12 h/12 h light/dark cycle, ∼24°C, ∼55% relative humidity) and mice had free access to tap water at all times. Animal trial 1: after an adaptation period to the facility and the intake of a liquid diet, female C57BL/6J mice, aged 6–8 weeks, were assigned randomly to the following groups (n = 6–8/group): C: mice fed a liquid control diet (69E% carbohydrate, 12E% lipids, 19E% protein; Ssniff, Soest, Germany), C+phosphatidylcholine (PC): mice fed a liquid control diet enriched with PC (12.5 mg/g diet), FrFC: mice fed a fructose-, fat-, and cholesterol-rich diet (60E% carbohydrate, 21E% lipids, 19E% protein with 50% wt/wt fructose and 0.16% wt/wt cholesterol; Ssniff), and FrFC+PC: mice fed a FrFC diet fortified with PC (12.5 mg/g diet) for 8 weeks. The sample size was determined based on previous findings.^30^ Phosphatidylcholine originating from egg lecithin (purity, 98%; Lipoid, Ludwigshafen, Germany) was used in all experiments. The dose of phosphatidylcholine was based on previous studies by other investigators.^55^ As detailed previously,^30^ the intake of the liquid diet was documented daily, and within dietary groups, mice received the amount of diet that was consumed the previous day by the group with the lowest food intake (pair-group). This pair-group had access to diet ad libitum. The body weight was monitored weekly and the food intake was monitored daily. Moreover, a glucose tolerance test was performed during week 7 of the feeding trial. For that purpose, mice were fasted for 6 hours. Blood glucose levels were determined before and 15, 30, 60, 90, and 120 minutes after the intraperitoneal glucose injection. Animal trial 2: male C57BL/6J mice were assigned randomly to the following groups (n = 7/group): C: mice fed a liquid control diet (69E% carbohydrates, 12E% fat, 19E% protein; Ssniff) or FrF: mice fed a liquid fructose- and fat-rich diet (60E% carbohydrates, 25E% fat, 15E% protein; Ssniff) for 7 weeks.

After 7–8 weeks of feeding, mice were anesthetized (intraperitoneally) with ketamine/xylazine in the morning, blood was taken from the portal vein, and mice then were killed by cervical dislocation. Hepatic tissue samples were collected in neutral-buffered formalin or immediately snap-frozen in liquid nitrogen and were stored in a −80°C freezer for further experiments. Killing and all following measurements were performed in a mixed order of groups.

### Cell Culture Experiments in J774A.1 Cells and Primary Human Monocytes

J774A.1 cells (DSMZ, Braunschweig, Germany) were cultured at 37°C in a humidified, 5% carbon dioxide atmosphere with Dulbecco’s modified Eagle medium (Pan-Biotech, Aidenbach, Germany) enriched with 1% penicillin/streptomycin (Pan-Biotech) and 10% fetal bovine serum (Pan-Biotech) upon reaching 80% confluency. Human primary monocytes isolated from buffy coats were isolated using density gradient centrifugation via the pluriSelect system (pluriSelect Life Science, Leipzig, Germany) and maintained in RPMI 1640 (Pan-Biotech) supplemented with 10% fetal bovine serum and 1% penicillin/streptomycin. In a first set of experiments, J774A.1 cells and human primary monocytes, respectively, were preincubated for 2 hours with medium containing either ±0.38 mmol/L phosphatidylcholine (Lipoid GmbH, Ludwigshafen, Germany), ±10 μmol/L of the PPARγ antagonist GW9662 (Sigma-Aldrich Chemie GmbH, Steinheim, Germany), or ±10 μmol/L of the PPARγ agonist pioglitazone (Santa Cruz Biotechnology, Inc, Heidelberg, Germany), or vehicle, followed by stimulation with 0 to 100 ng/mL LPS (serotype O55:B5; Sigma-Aldrich GmbH). In a second set of experiments, J774A.1 cells or human primary monocytes, respectively, were preincubated for 2 hours with medium containing either ±0.38 mmol/L phosphatidylcholine or ±1 to 10 μmol/L of an LRH-1 antagonist (1-[3’-(1-[2-(4-Morpholinyl)ethyl]-1H-pyrazol-3-yl)-3-biphenylyl]ethanon; Sigma-Aldrich Chemie GmbH) or vehicle, followed by a challenge with 0 to 100 ng/mL LPS. In each set of experiments medium was collected either after 6 hours (primary human monocytes) or after 18 hours (J774A.1 cells) and either immediately subjected to further analysis or stored at -80°C until further use. Furthermore, J774A.1 cells were lysed in TRItidy G (PanReac AppliChem, Darmstadt, Germany) and stored at -80°C for subsequent RNA isolation.

### Evaluation of Liver Damage and Inflammation and Blood Parameter of Liver Damage

Paraffin-embedded liver sections (4 μm) were stained with H&E (Sigma-Aldrich Chemie GmbH) to evaluate liver histology using NAS, as detailed by others.^56^ To assess hepatic fibrosis, liver sections were stained with Picrosirius red and counterstained with fast green (Sigma-Aldrich Chemie GmbH) as detailed previously.^57^ Neutrophil granulocytes were stained and counted in liver sections using a commercially available kit (naphthol AS-D chloroacetate kit; Sigma-Aldrich Chemie GmbH) as detailed previously,^58^ or were stained with Ly6G antibody (anti-Ly6G, #ab238132; Abcam, Cambridge, UK). In brief, antigen retrieval was performed and endogenous peroxidase activity was quenched by incubating sections with a peroxidase block (Dako Staining Solutions, Agilent Technologies, Vienna, Austria). Sections were blocked with 1% bovine serum albumin in phosphate-buffered saline with Tween 20 and incubated with a monoclonal antibody (anti-Ly6G, #ab238132; Abcam) for 1.5 hours at room temperature before incubating with secondary antibody for 20 minutes at room temperature (anti-rabbit; Dako Staining Solutions, Agilent Technologies). After staining with 3,3′-Diaminobenzidine chromogen solution (Dako Staining Solutions, Agilent Technologies) and counterstaining with hematoxylin (Sigma-Aldrich Chemie GmbH), sections were covered with a coverslip. Neutrophils and Ly6G-positive cells were counted and the mean of 8 microscopic fields was determined using a microscope (DFC 450 C; Leica, Wetzlar, Germany). Alanine aminotransferase activity in plasma was measured in a routine laboratory (Veterinary Medical University of Vienna, Vienna, Austria).

### Assessment of Arginase and MPO Activity and NO and Phosphatidylcholine Concentrations

Arginase and MPO activity and NO concentration in liver tissue were assessed as described previously.^30^^,^^59^ In brief, to determine arginase activity, liver tissue was homogenized in 50 mmol/L Tris-HCl, pH 7.5, containing 0.1% Triton X-100, 10 mmol/L MnCl_2_, and protease inhibitor cocktail (Sigma-Aldrich Chemie GmbH), and liver tissue was homogenized in phosphate buffer for measuring MPO activity. NO concentration in liver tissue as well as in cell culture supernatant was measured using a commercially available Griess reagent assay (#G2930; Promega, Mannheim, Germany). Phosphatidylcholine concentration in liver tissue was measured using a commercially available assay following the manufacturer’s instructions (#MAK049; Sigma-Aldrich Chemie GmbH).

### Immunohistochemical Staining of PPARγ2

To determine in which cells PPARγ2 is expressed predominately in liver, liver sections were stained with a specific monoclonal primary antibody. In brief, after antigen retrieval and peroxidase blocking, liver sections were stained with a monoclonal primary antibody against PPARγ2 (#sc-390740; Santa Cruz Biotechnology) overnight at 4°C and biotin-labeled secondary antibody for 30 minutes and enzyme-labeled streptavidin (Dako Staining Solutions, Agilent Technologies) for 30 minutes, respectively. Representative pictures were taken using a microscope with an integrated camera (DM4000 B LED; Leica).

### Assessment of TLR4 Ligand Concentration in Plasma

Commercially available reporter gene assays with TLR4-transfected HEK293 cells were used to determine the levels of TLR4 ligands in plasma, following the manufacturer’s instructions (InvivoGen, Toulouse, France) as described previously in detail.^60^

### Enzyme-Linked Immunosorbent Assays, RNA Isolation, and Real-Time Polymerase Chain Reaction

Using commercially available enzyme-linked immunosorbent assay kit concentrations of TNF-α (DuoSet; R&D Systems, Inc, Minneapolis, MN) were analyzed in cell culture supernatant following the manufacturer’s instructions. RNA from liver tissue and cells was extracted using TRItidy G (PanReac AppliChem). After measuring concentration of RNA, complementary DNA was synthetized with a reverse-transcription system (Promega GmbH). Using primers listed in Table 3, real-time polymerase chain reaction was performed. In female mice, expression of the respective genes was normalized to 18S and glyceraldehyde-3-phosphate dehydrogenase, of which a geometric mean was calculated as previously described.^31^^,^^61^ In male mice, expression of genes was normalized to the geometric mean of 18S, β-actin, and glyceraldehyde-3-phosphate dehydrogenase.

**Table 3:** Primer Sequences Used for Real-Time Polymerase Chain Reaction

	Forward, 5’- 3’	Reverse, 5’-3’
*18S*	GTAACCCGTTGAACCCCATT	CCATCCAATCGGTAGTAGCG
*β-actin*	GGCTCCTAGCACCATGAA	AGCCACCGATCCACACAGA
*Apob*	TCACCATTTGCCCTCAACCT	CAGGTCAACATCGGCAATCA
*Gapdh*	AAAAGGGTCATCATCTCCGC	AGCCCTTCCACAATGCCAAA
*Lrh1 (Nr5a2)*	ACCTGGCTACGTTGACTCTT	TGGATGTAACCCGCTGTGTT
*Pparg1*	AACGTGAAGCCCATCGAGGA	CTGCACGTGCTCTGTGACGA
*Pparg2*	TCGCTGATGCACTGCCTATG	GAGAGGTCCACAGAGCTGATT
*Tlr4*	AGCCATTGCTGCCAACATCA	GCTGCCTCAGCAGGACTTC
*Tnfa*	CAGCCAACCAGGCAGGTTCT	CCTGCCACAAGCAGGAATGA

### Western Blot Analysis

Liver tissue was homogenized either in lysis buffer (1 mol/L HEPES, 1 mol/L MgCl_2_, 2 mol/L KCl, and 1 mol/L dithiothreitol) containing protease and phosphatase inhibitors mix (Sigma-Aldrich Chemie GmbH) to obtain cytosolic protein lysates. To obtain total protein, liver tissue was homogenized either in RIPA buffer (20 mmol/L 3-[N-morpholino] propanesulfonic acid, 150 mmol/L NaCl, 1 mmol/L ethylenediaminetetraacetic acid, 1% Nonidet P-40, and 0.1% sodium dodecyl sulfate) containing protease and phosphatase inhibitor cocktails (Sigma-Aldrich Chemie GmbH) or in TRItidy G (PanReac AppliChem) according to the manufacturer’s instructions. Protein lysates then were separated in a sodium dodecyl sulfate–polyacrylamide gel and transferred to a Hybond-P polyvinylidene difluoride membrane (Bio-Rad Laboratories, Hercules, CA). Membranes were incubated further with specific primary antibodies (phosphorylated IkBα [Ser32/36] [#9246], total IkBα [#9242], TNF-α [#3707]; all Cell Signaling Technology, Inc; PPARγ2 [#sc-390740], and β-actin [#sc-47778]; Santa Cruz Biotechnology; ApoB [#MSB127308]; MyBioSource, Inc), followed by incubation with the respective secondary antibody (Cell Signaling Technology, Inc). For the detection of protein bands, the Super Signal Western Dura kit (Thermo Fisher Scientific, Waltham, MA) was used, and densitometric analyses were performed using the ChemiDoc XRS System (Bio-Rad Laboratories) as detailed previously.^58^

### Statistical Analysis

Using PRISM (version 7.03; GraphPad Software, San Diego, CA), a Grubb’s outlier test was performed for all measurements before further statistical analysis. To analyze differences between 2 groups, an unpaired Student *t* test was used. To determine statistical differences between 3 or more groups, a 1-way analysis of variance and a 2-way analysis of variance, respectively, were applied, followed by the Tukey post hoc test. The Bartlett test was used to assess homogeneity of variance, and in case of inhomogeneity of variances, data were log-transformed. A *P* value less than .05 was defined as significant. All data are presented as means ± SD, or as box and whisker plots.

## References

[1] Rinella M.E., Lazarus J.V., Ratziu V. (2024). A multi-society Delphi consensus statement on new fatty liver disease nomenclature. Ann Hepatol.

[2] Younossi Z.M., Golabi P., Paik J.M. (2023). The global epidemiology of nonalcoholic fatty liver disease (NAFLD) and nonalcoholic steatohepatitis (NASH): a systematic review. Hepatology.

[3] Farrell G.C., Larter C.Z. (2006). Nonalcoholic fatty liver disease: from steatosis to cirrhosis. Hepatology.

[4] Mirizzi A., Franco I., Leone C.M. (2019). Effects of some food components on non-alcoholic fatty liver disease severity: results from a cross-sectional study. Nutrients.

[5] Petta S., Di Marco V., Pipitone R.M. (2018). Prevalence and severity of nonalcoholic fatty liver disease by transient elastography: genetic and metabolic risk factors in a general population. Liver Int.

[6] Hughey C.C., Puchalska P., Crawford P.A. (2022). Integrating the contributions of mitochondrial oxidative metabolism to lipotoxicity and inflammation in NAFLD pathogenesis. Biochem Biophys Acta Mol Cell Biol Lipids.

[7] Rivera C.A., Adegboyega P., van Rooijen N. (2007). Toll-like receptor-4 signaling and Kupffer cells play pivotal roles in the pathogenesis of non-alcoholic steatohepatitis. J Hepatol.

[8] Rohr M.W., Narasimhulu C.A., Rudeski-Rohr T.A. (2020). Negative effects of a high-fat diet on intestinal permeability: a review. Adv Nutr.

[9] Brandt A., Hernández-Arriaga A., Kehm R. (2019). Metformin attenuates the onset of non-alcoholic fatty liver disease and affects intestinal microbiota and barrier in small intestine. Sci Rep.

[10] Baumann A., Burger K., Brandt A. (2022). GW9662, a peroxisome proliferator-activated receptor gamma antagonist, attenuates the development of non-alcoholic fatty liver disease. Metabolism.

[11] Brandl K., Schnabl B. (2017). Intestinal microbiota and nonalcoholic steatohepatitis. Curr Opin Gastroenterol.

[12] Deng K.-Q., Huang X., Lei F. (2022). Role of hepatic lipid species in the progression of nonalcoholic fatty liver disease. Am J Physiol Cell Physiol.

[13] van der Veen J.N., Kennelly J.P., Wan S. (2017). The critical role of phosphatidylcholine and phosphatidylethanolamine metabolism in health and disease. Biochim Biophys Acta.

[14] Zwolak A., Szuster-Ciesielska A., Daniluk J. (2015). Hyperreactivity of blood leukocytes in patients with NAFLD to ex vivo lipopolysaccharide treatment is modulated by metformin and phosphatidylcholine but not by alpha ketoglutarate. PLoS One.

[15] Niebergall L.J., Jacobs R.L., Chaba T. (2011). Phosphatidylcholine protects against steatosis in mice but not non-alcoholic steatohepatitis. Biochim Biophys Acta.

[16] Ling J., Chaba T., Zhu L.-F. (2012). Hepatic ratio of phosphatidylcholine to phosphatidylethanolamine predicts survival after partial hepatectomy in mice. Hepatology.

[17] Li Z., Agellon L.B., Allen T.M. (2006). The ratio of phosphatidylcholine to phosphatidylethanolamine influences membrane integrity and steatohepatitis. Cell Metab.

[18] Feng T.-T., Yang X.-Y., Hao S.-S. (2020). TLR-2-mediated metabolic reprogramming participates in polyene phosphatidylcholine-mediated inhibition of M1 macrophage polarization. Immunol Res.

[19] Durante W., Johnson F.K., Johnson R.A. (2007). Arginase: a critical regulator of nitric oxide synthesis and vascular function. Clin Exp Pharmacol Physiol.

[20] Jacobs R.L., Devlin C., Tabas I. (2004). Targeted deletion of hepatic CTP:phosphocholine cytidylyltransferase alpha in mice decreases plasma high density and very low density lipoproteins. J Biol Chem.

[21] Mitzscherling K., Volynets V., Parlesak A. (2009). Phosphatidylcholine reverses ethanol-induced increase in transepithelial endotoxin permeability and abolishes transepithelial leukocyte activation. Alcohol Clin Exp Res.

[22] Parlesak A., Schaeckeler S., Moser L. (2007). Conjugated primary bile salts reduce permeability of endotoxin through intestinal epithelial cells and synergize with phosphatidylcholine in suppression of inflammatory cytokine production. Crit Care Med.

[23] Sakai J., Cammarota E., Wright J.A. (2017). Lipopolysaccharide-induced NF-κB nuclear translocation is primarily dependent on MyD88, but TNFα expression requires TRIF and MyD88. Sci Rep.

[24] Viatour P., Merville M.-P., Bours V. (2005). Phosphorylation of NF-κB and IκB proteins: implications in cancer and inflammation. Trends Biochem Sci.

[25] Yin H., Liu Y., Yue H. (2022). DHA- and EPA-enriched phosphatidylcholine suppress human lung carcinoma 95D cells metastasis via activating the peroxisome proliferator-activated receptor γ. Nutrients.

[26] Wang Y., Nakajima T., Gonzalez F.J. (2020). PPARs as metabolic regulators in the liver: lessons from liver-specific PPAR-null mice. Int J Mol Sci.

[27] Lee J.M., Lee Y.K., Mamrosh J.L. (2011). Antidiabetic actions of a phosphatidylcholine ligand for nuclear receptor LRH-1. Nature.

[28] Sun Y., Demagny H., Schoonjans K. (2021). Emerging functions of the nuclear receptor LRH-1 in liver physiology and pathology. Biochim Biophys Acta.

[29] Duric M., Sivanesan S., Bakovic M. (2012). Phosphatidylcholine functional foods and nutraceuticals: a potential approach to prevent non-alcoholic fatty liver disease. Eur J Lipid Sci Technol.

[30] Sánchez V., Brandt A., Jin C.J. (2021). Fortifying butterfat with soybean oil attenuates the onset of diet-induced non-alcoholic steatohepatitis and glucose intolerance. Nutrients.

[31] Brandt A., Rajcic D., Jin C.J. (2020). Fortifying diet with rapeseed oil instead of butterfat attenuates the progression of diet-induced non-alcoholic fatty liver disease (NAFLD) and impairment of glucose tolerance. Metabolism.

[32] Iwakiri Y., Kim M.Y. (2015). Nitric oxide in liver diseases. Trends Pharmacol Sci.

[33] Palmieri E.M., Gonzalez-Cotto M., Baseler W.A. (2020). Nitric oxide orchestrates metabolic rewiring in M1 macrophages by targeting aconitase 2 and pyruvate dehydrogenase. Nat Commun.

[34] Rath M., Müller I., Kropf P. (2014). Metabolism via arginase or nitric oxide synthase: two competing arginine pathways in macrophages. Front Immunol.

[35] Sherriff J.L., O'Sullivan T.A., Properzi C. (2016). Choline, its potential role in nonalcoholic fatty liver disease, and the case for human and bacterial genes. Adv Nutr.

[36] Zhao F., Li R., Liu Y. (2023). Perspectives on lecithin from egg yolk: extraction, physicochemical properties, modification, and applications. Front Nutr.

[37] Osipova D., Kokoreva K., Lazebnik L. (2022). Regression of liver steatosis following phosphatidylcholine administration: a review of molecular and metabolic pathways involved. Front Pharmacol.

[38] Noga A.A., Vance D.E. (2003). Insights into the requirement of phosphatidylcholine synthesis for liver function in mice. J Lipid Res.

[39] Noga A.A., Vance D.E. (2003). A gender-specific role for phosphatidylethanolamine N-methyltransferase-derived phosphatidylcholine in the regulation of plasma high density and very low density lipoproteins in mice. J Biol Chem.

[40] Noga A.A., Zhao Y., Vance D.E. (2002). An unexpected requirement for phosphatidylethanolamine N-methyltransferase in the secretion of very low density lipoproteins. J Biol Chem.

[41] Spruss A., Kanuri G., Wagnerberger S. (2009). Toll-like receptor 4 is involved in the development of fructose-induced hepatic steatosis in mice. Hepatology.

[42] Ai R., Xu J., Ji G. (2022). Exploring the phosphatidylcholine in inflammatory bowel disease: potential mechanisms and therapeutic interventions. Curr Pharm Des.

[43] Chen M., Huang H., Zhou P. (2019). Oral phosphatidylcholine improves intestinal barrier function in drug-induced liver injury in rats. Gastroenterol Res Pract.

[44] Jacobs M.D., Harrison S.C. (1998). Structure of an IkBalpha/NF-kB complex. Cell.

[45] Vaure C., Liu Y. (2014). A comparative review of Toll-like receptor 4 expression and functionality in different animal species. Front Immunol.

[46] Ishikado A., Nishio Y., Yamane K. (2009). Soy phosphatidylcholine inhibited TLR4-mediated MCP-1 expression in vascular cells. Atherosclerosis.

[47] Won T.J., Nam Y., Lee H.S. (2013). Injection of phosphatidylcholine and deoxycholic acid regulates gene expression of lipolysis-related factors, pro-inflammatory cytokines, and hormones on mouse fat tissue. Food Chem Toxicol.

[48] Liu Y., Tian Y., Cai W. (2021). DHA/EPA-enriched phosphatidylcholine suppresses tumor growth and metastasis via activating peroxisome proliferator-activated receptor γ in Lewis lung cancer mice. J Agric Food Chem.

[49] Hou Y., Moreau F., Chadee K. (2012). PPARγ is an E3 ligase that induces the degradation of NFκB/p65. Nat Commun.

[50] Wang H., Zhao Y., Pan Y. (2023). Inhibition of phospholipase D1 ameliorates hepatocyte steatosis and non-alcoholic fatty liver disease. JHEP Rep.

[51] Benod C., Carlsson J., Uthayaruban R. (2013). Structure-based discovery of antagonists of nuclear receptor LRH-1. J Biol Chem.

[52] Sahini N., Borlak J. (2016). Genomics of human fatty liver disease reveal mechanistically linked lipid droplet–associated gene regulations in bland steatosis and nonalcoholic steatohepatitis. Transl Res.

[53] Inoue M., Ohtake T., Motomura W. (2005). Increased expression of PPARgamma in high fat diet-induced liver steatosis in mice. Biochem Biophys Res Commun.

[54] Zizzo G., Cohen P.L. (2015). The PPAR-γ antagonist GW9662 elicits differentiation of M2c-like cells and upregulation of the MerTK/Gas6 axis: a key role for PPAR-γ in human macrophage polarization. J Inflamm (Lond).

[55] Tandy S., Chung R.W.S., Kamili A. (2010). Hydrogenated phosphatidylcholine supplementation reduces hepatic lipid levels in mice fed a high-fat diet. Atherosclerosis.

[56] Kleiner D.E., Brunt E.M., Van Natta M. (2005). Design and validation of a histological scoring system for nonalcoholic fatty liver disease. Hepatology.

[57] Baumann A., Hernández-Arriaga A., Brandt A. (2021). Microbiota profiling in aging-associated inflammation and liver degeneration. Int J Med Microbiol.

[58] Spruss A., Kanuri G., Stahl C. (2012). Metformin protects against the development of fructose-induced steatosis in mice: role of the intestinal barrier function. Lab Invest.

[59] Rajcic D., Baumann A., Hernandez-Arriaga A. (2021). Citrulline supplementation attenuates the development of non-alcoholic steatohepatitis in female mice through mechanisms involving intestinal arginase. Redox Biol.

[60] Jung F., Burger K., Staltner R. (2021). Markers of intestinal permeability are rapidly improved by alcohol withdrawal in patients with alcohol-related liver disease. Nutrients.

[61] Vandesompele J., De Preter K., Pattyn F. (2002). Accurate normalization of real-time quantitative RT-PCR data by geometric averaging of multiple internal control genes. Genome Biol.

